# Vascularization ability of glioma stem cells in different three-dimensional microenvironments

**DOI:** 10.1093/rb/rbad094

**Published:** 2023-10-27

**Authors:** Xuanzhi Wang, Tao Xu, Chaoshi Niu

**Affiliations:** Department of Neurosurgery, The First Affiliated Hospital of USTC, Division of Life Sciences and Medicine, University of Science and Technology of China, Hefei, Anhui, 230036, China; Department of Neurosurgery, Sichuan Provincial People’s Hospital, University of Electronic Science and Technology of China, Chengdu 610072, People’s Republic of China; Center for Bio-intelligent Manufacturing and Living Matter Bioprinting, Research Institute of Tsinghua University in Shenzhen, Tsinghua University, Shenzhen 518057, People’s Republic of China; Department of Neurosurgery, The First Affiliated Hospital of USTC, Division of Life Sciences and Medicine, University of Science and Technology of China, Hefei, Anhui, 230036, China

**Keywords:** 3D bioprinting, xenograft tumors, GSC-laden hydrogel scaffold, glioblastoma, angiogenesis

## Abstract

Glioblastoma (GBM) is among the most common and aggressive adult central nervous system tumors. One prominent characteristic of GBM is the presence of abnormal microvessels. A significant correlation between angiogenesis and prognosis has been observed. Accurately reconstructing this neovascularization and tumor microenvironment through personalized *in vitro* disease models presents a significant challenge. However, it is crucial to develop new anti-angiogenic therapies for GBM. In this study, 3D bioprinted glioma stem cell (GSC)-laden hydrogel scaffolds, hybrid GSC hydrogels and cell-free hydrogel scaffolds were manufactured to investigate the vascularization ability of GSCs in varying 3D microenvironments. Our results demonstrated that the bioactivity of GSCs in the 3D bioprinted GSC-laden hydrogel scaffold was preferable and stable, and the amounts of vascular endothelial growth factor A and basic fibroblast growth factor were the highest in the microenvironment. When the three different models were co-cultured with human umbilical vein endothelial cells, the expression of angiogenesis-related markers was the most abundant in the bioprinted GSC-laden hydrogel scaffold. Additionally, xenograft tumors formed by bioprinted GSC-laden hydrogel scaffolds more closely resembled human gliomas regarding color, texture and vascularization. Notably, in xenograft tumors derived from 3D bioprinted GSC-laden hydrogel scaffolds, the number of human CD105^+^ cells was significantly higher, and human endothelial vascular lumen-like structures were observed. This indicates that the 3D bioprinted GSC-laden hydrogel scaffold is a suitable model for mimicking the glioma microenvironment and studying tumor angiogenesis.

## Graphical Abstract

**Figure:**
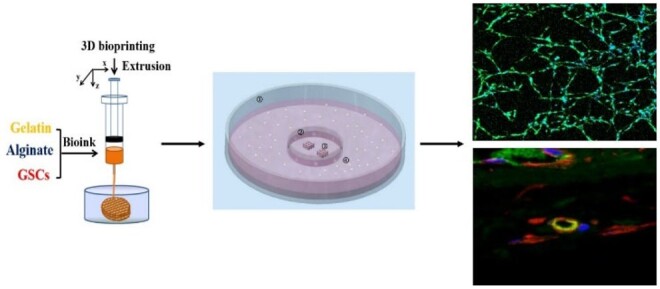

## Introduction

Glioblastoma (GBM) is the most common and aggressive primary brain cancer in adults, with a high recurrence and mortality rate owing to the difficulty of complete surgical resection and resistance to conventional chemotherapy/radiotherapy [1]. GBM is a highly vascularized tumor whose growth depends on the formation of new blood vessels [2]. Tumor proliferation induces angiogenesis, and tumor cells invade the periphery through blood vessels [3]. Therefore, an in-depth study on the mechanism of angiogenesis in GBM is of profound value for its treatment. In particular, understanding the mechanism of neovascularization in GBM is crucial for anti-angiogenic tumor therapy. Therefore, GBM models must be constructed *in vitro* or *in vivo* to investigate tumor angiogenesis patterns.

Recently, an increasing number of 3D models have been used to study the angiogenesis mechanisms of GBM [4]. Several studies have shown that 3D microenvironments enhance cell–cell and cell–matrix interactions and promote cell proliferation, migration, invasion and gene expression *in vivo* [5, 6]. Therefore, an encouraging tumor angiogenesis model is necessary to study GBM neovascularization. Advances in 3D bioprinting technology have inspired researchers to develop 3D tumor models that are ‘printed’ *in vitro* to investigate the mechanisms of tumor angiogenesis [7]. Nguyen designed a 3D GBM angiogenesis model based on the microwells of a gelatin methacrylate hydrogel *in vitro*, on which GBM spheroids were inoculated and co-cultured with the surrounding endothelial cells. Endothelial cells migrated to the glioma cells and formed microtubules [8]. However, these GBM spheroids were implanted in a 3D hydrogel model, which did not conform to the distribution and microenvironmental composition of tumor cells *in vivo*. Forrest reported that glioma cells cultured on 3D chitosan-alginate scaffolds had a stronger ability to form CD31-positive cells and caused more significant angiogenesis in nude mice than that attributable to traditional 2D cultures [9]. However, these glioma cells were transplanted into nude mice using the traditional 2D model. Noisa’s study found that GBM cells cultured on 3D calcium alginate scaffolds enhanced vascularization potential, and the expression of tumor angiogenesis biomarkers was higher than that in 2D cells. However, glioma cells implanted on the scaffold were unevenly distributed, and there was insufficient extracellular matrix around them to mimic the tumor microenvironment *in vivo* [10]. Tatla *et al*. introduced a 3D vascularized GBM-like tumor model *in vitro* to study tumor angiogenesis. This 3D model consisted of a fibrin gel containing the GBM and endothelial cells. GBM cells encapsulated in hydrogels promoted endothelial cell sprouting via secreting vascular growth factors [11]. Our previous studies have shown that 3D bioprinted glioma stem cells (GSCs) can secrete the vascular endothelial growth factor (VEGF) and form tubule-like structures *in vitro* [4]. According to previous reports, glioma cells in a 3D microenvironment exhibit good biological activity and are involved in tumor angiogenesis. However, which 3D microenvironment glioma cells have a stronger vascularization ability is yet to be determined.

The aforementioned advantages made us consider the advantages of 3D tumor models constructed by bioprinting. In this study, 3D bioprinted GSC-laden hydrogel scaffolds, hybrid GSC hydrogels and cell-free hydrogel scaffolds were developed to study glioma angiogenesis. Specifically, a personalized 3D co-culture microenvironment was constructed. Briefly, the biological properties of glioma cells in different 3D models were analyzed, and the vascularization capacity of endothelial cells in different 3D microenvironments was evaluated. More importantly, the angiogenesis mechanism of xenograft tumors formed using different 3D tumor models was investigated.

The rest of this article is organized as follows. The next section outlines the materials and methods used. Then, the results and discussions of the study are presented. The final section concludes the study.

## Materials and methods

### Cell culture

The GSC line GSC23, of human origin, was grown in a culture medium composed of Dulbecco’s Modified Eagle Medium/Nutrient Mixture F-12 (DMEM/F-12), enhanced with 20 ng/ml basic fibroblast growth factor (bFGF), 20 ng/ml epidermal growth factor and B27 supplement (50×) (all obtained from Gibco, Carlsbad, CA, USA). Human umbilical vein endothelial cells (HUVECs, Passages 3–4) were procured from the Cell Bank of the Chinese Academy of Sciences (Shanghai, China) and maintained in DMEM enriched with 10% fetal bovine serum (FBS) (sourced from Gibco). Both cell types were grown in an environment of 37°C and 5% CO_2_. The culture medium was refreshed every 48–72 h.

### Bioprinting material preparation

Gamma radiation (15 Gy) was employed for the sterilization of sodium alginate (Sigma, A0682) and gelatin (Sigma, Type A, V900863), which were then dissolved in a 0.9% sodium chloride solution (w/v) at concentrations of 2% and 20% (w/v), respectively. For the 3D bioprinting process, GSC23 cells (4 × 10^5^/ml) were enzymatically digested and resuspended in 2% sodium alginate and 20% gelatin solutions to reach a final concentration of 10% gelatin, 1% sodium alginate and 2 × 10^5^/ml GSC23 cells.

### Design and creation of co-culture dishes

The co-culture dishes used in this study were designed and fabricated by individuals and comprised two concentric circles: an exterior and interior dish. The interior dish communicated with the exterior dish through side holes.

### 3D bioprinted GSC model

GSC models were generated using an extrusion bioprinter (Medprin Regenerative Medical Technologies Co., Ltd., Guangzhou, China) following a previously documented method [12]. In short, circular grid scaffolds were fabricated with a diameter of 15 mm and a thickness of 1 mm. Following bioprinting, the cell-laden scaffolds were crosslinked using a 3% (w/v) CaCl_2_ solution for 3 minutes. This research proposed both cell-free hydrogel scaffolds and hybrid cell hydrogels. Cell-free hydrogel scaffolds were created using the same method as the 3D bioprinted cell-laden hydrogel scaffolds, excluding the addition of GSCs during the printing process. The GSCs were directly blended into the ready printing material, injected into a sterile mold measuring 15 mm in diameter and 1 mm in height and cross-linked to create a circular hybrid cell hydrogel.

### Creation of 3D GSC tumor model co-cultured with HUVEC

3D bioprinted GSC-laden hydrogel scaffolds, cell-free hydrogel scaffolds and hybrid GSC hydrogels crafted using varying methods were co-cultured with HUVEC in custom-made culture dishes. In this research, 3D models were situated in the interior dish, 5 × 10^4^ HUVECs were introduced into the exterior dish, and both dishes were cultivated in DMEM enriched with 10% FBS (without exogenous vascular growth factor).

### Analysis of the biological activity of GSCs in 3D models

A fluorescent live/dead assay kit (Keygen, Nanjing, China) was utilized to assess cellular viability. In short, 3D bioprinted GSC-laden hydrogel scaffolds and hybrid GSC hydrogels were subjected to staining by soaking them in a blended solution of 8 μM propidium iodide and 2 μM calcein-AM for a period of 10 min, devoid of light exposure and at a temperature of 37°C. Following this, the samples underwent a triple rinse process using phosphate-buffered saline (PBS), after which images were procured using a fluorescence microscope. Viable cells are depicted in green, and deceased cells appear in red. The survival rate was computed using this equation: survival rate (%) = (quantity of live cells/total cell count) × 100. Both live and dead cells within each sample (*n* = 3) were quantified in five arbitrary fields at ×200 magnification.

To visualize the morphological traits and distribution patterns of the GSCs in the 3D tumor models, samples were initially fixed using 4% paraformaldehyde (Keygen, Nanjing, China) for 24 h and subsequently encased in paraffin. These samples were sliced into sections of 5 μm thickness and treated with hematoxylin and eosin (HE) following the established protocol [13]. The Leica DM500 microscope was used to capture images. Likewise, the 3D models underwent overnight fixation at 4°C using 2.5% glutaraldehyde, followed by dehydration through a graded ethanol series (70%, 80%, 90%, 95%, 100% and 100%) for 30 min each. Critical point drying was performed using a carbon dioxide critical point dryer (LEICA, EM CPD300). After sputter coating with Pt, images were taken using an ULTRA 55 scanning electron microscope (SEM) (ZEISS, Germany).

The VEGFA and bFGF concentrations present in the co-culture dish supernatants were determined employing sandwich enzyme immunoassay kits (VEGFA-ELISA kit, bFGF-ELISA kit, Donglin Science & Technology, Wuxi, China) following the guidelines provided by the manufacturer. In brief, 100 μl of the supernatants from each sample and standard solution at various concentrations were added to individual wells of microtiter plates and incubated for 2 h at 37°C. The liquid was subsequently replaced with 100 μl of detection reagent A working solution, followed by a 1-hour incubation at 37°C. Thereafter, 100 μl of detection reagent B working solution was added, and incubation continued for another hour at 37°C. Afterward, 90 μl of the substrate solution was added to each well and incubated at 37°C for 20 min in light-deprived conditions. Approximately 50 μl of stop solution was added, after which optical density (OD) readings were immediately taken at a wavelength of 450 nm (BioTek ELX800, VT, USA). A standard curve was plotted based on the ODs of the standard solutions, facilitating the calculation of VEGFA and bFGF concentrations.

### Analysis of cell proliferation and vascularization capability of co-cultured HUVEC *in vitro*

The morphological shifts in HUVECs in the outer dishes after co-culturing with diverse 3D tumor models were noted on Days 1, 3, 5 and 7. Alamar Blue reagent was employed to monitor HUVEC cell proliferation *in vitro*. Concisely, a working solution prepared from the fresh medium was introduced to each co-cultured HUVEC group and left to incubate at 37°C for 2 h. The incubated assay solution was transferred into a 96-well plate at 100 μl per well, and the OD values were read at 570 and 630 nm wavelengths. OD values of each group were normalized to Day 1 for charting and statistical assessment. The HUVECs were fixed with 4% paraformaldehyde at room temperature for 20 min, permeabilized with 0.1% Triton X-100 solution for 5 min, then stained with Phalloidin working solution for 30 min at room temperature in a dark setting, and rinsed three times with PBS. Following this, the nuclei were restained with 4′,6-diamidino-2-phenylindole for 10 min and images were captured with a laser confocal microscope (ZEISS, Germany, LSM800). Image-J software (Rawak Software, Inc., Germany) was applied to quantify the number of tubules represented by closed loops of HUVECs to quantify the formation of tubule-like structures. In brief, the Angiogenesis Analyze tool and Network Analysis Menu of Image J software were executed. The number of tubule-like structures was utilized to indicate the vascularization capability of the HUVECs [14].

HUVECs were harvested after 7 days of co-culture with various 3D models. qRT-PCR and western blot were applied to detect the gene and protein expression of markers related to vascular genesis in endothelial cells, including CD31, CD105, von Willebrand factor (vWF), VEGFR2 and FGFR1. In brief, HUVECs were thoroughly dissociated using Trizol (Invitrogen, 15596–026), and total RNA was extracted according to the manufacturer’s instructions. The ImProm-IITM reverse transcription system (Promega, A3800) was used to convert mRNA into cDNA. DNA transcription was performed using the SYBR Green qPCR Super Mix, with glyceraldehyde 3-phosphate dehydrogenase (GAPDH) serving as an internal standard. Relative gene expression was computed using the 2^−ΔΔCt^ method. HUVECs were collected and lysed for western blotting with RIPA buffer (KeyGEN BioTECH, Nanjing, China). The total protein concentration was evaluated using a Bicinchoninic acid protein assay kit (Keygen BioTECH, Nanjing, China) and transferred onto Immobilon-PPVDF membranes (Millipore, Billerica, MA, USA). Afterward, the protein was blocked with a 5% skim milk solution for 1 h and incubated overnight at 4°C with primary antibodies anti-VEGFR2 (ab134191), anti-CD31 (ab9498), anti-CD105 (ab252345), anti-vWF(ab201336) and anti-FGFR1 (ab173305), all sourced from Abcam. GAPDH was used as an internal reference. The gray values of the protein bands were measured using Image-J software, and the level of the target protein was normalized to the internal reference for charting and statistical analysis.

### Establishment of xenograft tumor model

The construction and execution of all animal tests received approval from the Institutional Ethics Board of the First Affiliated Hospital of the USTC (ethical approval number: 2022-N(A)-096). BALB/c hairless mice (aged 4–6 weeks) were anesthetized via intraperitoneal injection of 1% pentobarbital sodium solution (30 mg/kg). A cut was made on the dorsal skin, and the subcutaneous tissue was parted. Following this, the 3D bioprinted GSC-laden hydrogel scaffolds, cell-free hydrogel scaffolds and hybrid GSC hydrogels, all cultured *in vitro* for 7 days, were implanted into the subcutaneous tissue of the mice, and the skin cut was stitched up. The nude mice were raised separately after surgery, and the skin cut was frequently disinfected.

### Histological examination of xenografts

The subcutaneous xenografts from all mouse groups were extracted in the sixth week following transplantation, and the gross morphology and texture of the xenografts were visually assessed. The xenografts were preserved with 4% paraformaldehyde at 4°C overnight. HE and immunohistochemical staining (CD31, VEGFA, CD105) were performed according to the kit guidelines. HE staining was used to determine the existence of a hydrogel within the xenograft, the absence of the hydrogel grid structure, and the presence of tumor cells within the hydrogel. Immunohistochemical staining for CD31 (anti-human/mouse CD31 (ab28364), Abcam) and VEGFA (anti-human/mouse VEGFA (ab52917), Abcam) was employed to assess neovascularization in the xenograft. CD105 (anti-human CD105 (ab114052) and anti-human/mouse CD105 (ab107595), both from Abcam) were used to evaluate the vascular component of the xenografts. In this research, the source of neovascularization in the xenografts was examined by double immunofluorescence staining for vWF (rabbit anti-human vWF (ab154193), Abcam) and glial fibrillary acidic protein (GFAP) (mouse anti-human GFAP (MAB2594), R&D Biosystems), following the manufacturer’s guidance.

### Density of microvessels

In this research, CD105 immunohistochemical staining was employed to measure the microvessel density (MVD) of the xenografts [15]. Briefly, regions with the greatest neovascularization were identified at low magnification (40× magnification). Images were captured at 200× magnification (Olympus IX51 microscope, 0.74 mm^2^/field). The Image-J software was used to compute the number of microvessels. Any brown endothelial cell or cell cluster was considered a single countable microvessel. Five separate regions of CD105-positive cells or clusters were analyzed. MVD was defined as the number of microvessels seen in a 200× field (0.74 mm^2^).

### Statistical assessment

The experimental data were presented as the mean ± standard deviation, and GraphPad Prism 7.0 software was used for data analysis. The Student’s t-test was employed to compare means between the two groups. Two-way ANOVA (Analysis of Variance) and Bonferroni *post hoc* test were used to compare means among multiple groups. A value of *P ≤ 0.05, **P ≤ 0.01 and ***P ≤ 0.001 was considered to indicate statistical significance.

## Results

### 3D GSCs model co-cultured with HUVECs

**Figure 1.**
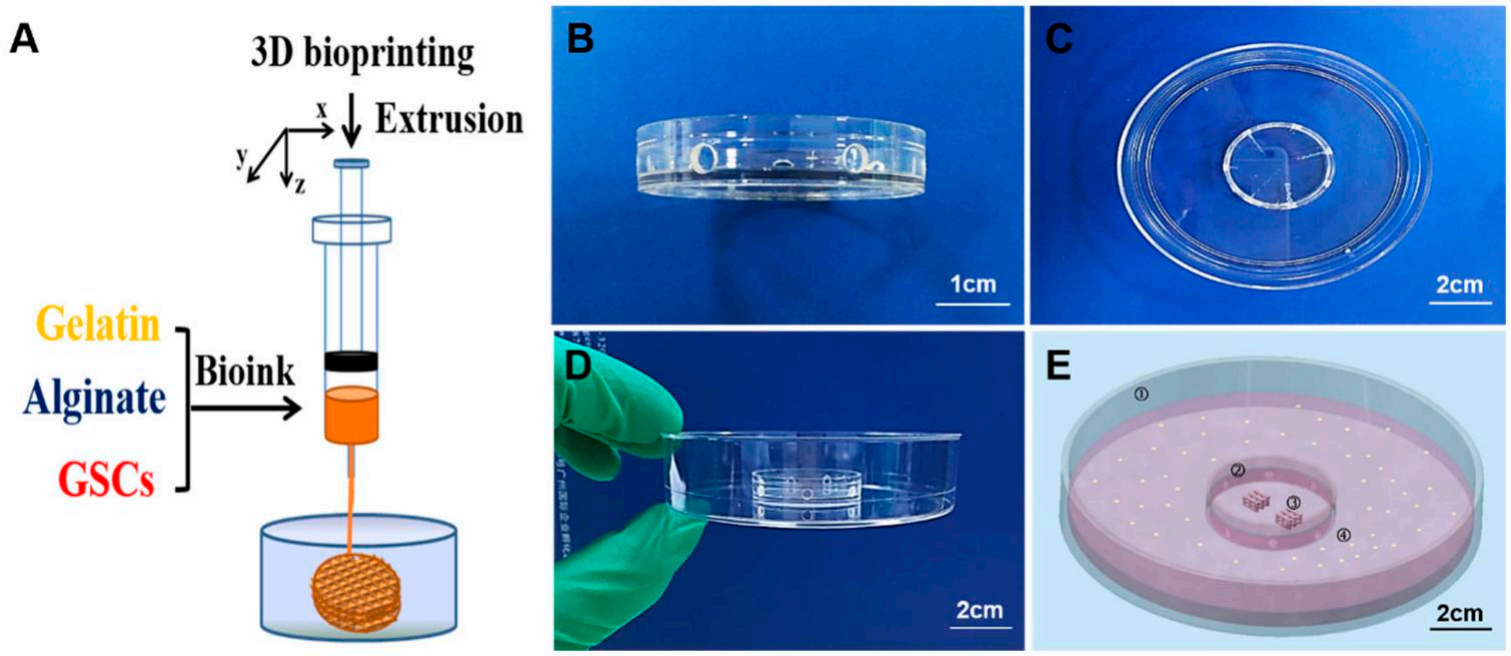
Establishment of co-culture system. (**A**) The schematic diagram of 3D bioprinting. (**B**–**D**) Manufacture of co-culture dishes. (**E**) 3D GSCs model co-cultured with HUVECs (①: outer dish; ②: inner dish; ③: 3D tumor models; ④: HUVECs).

Figure 1A shows a schematic of the bioprinted 3D GSCs model. As shown in Figure 1B–D, the materials of the co-culture dishes were all made of polystyrene, in which the diameter of the outer dish was 10 cm and that of the inner dish was 4 cm. Eight round holes with a diameter of 2.5 mm were evenly distributed on the side wall of the inner dish to ensure that the media in the inner and outer dishes could diffuse with each other. In this study, two 3D tumor models were placed in each inner dish, and 5 × 10^4^ HUVECs were inoculated into the outer dish. DMEM culture medium containing 10% FBS (without exogenous vascular growth factor) was used in both the inner and outer dishes (Figure 1E).

### Bioactivity of GSCs in 3D model

**Figure 2.**
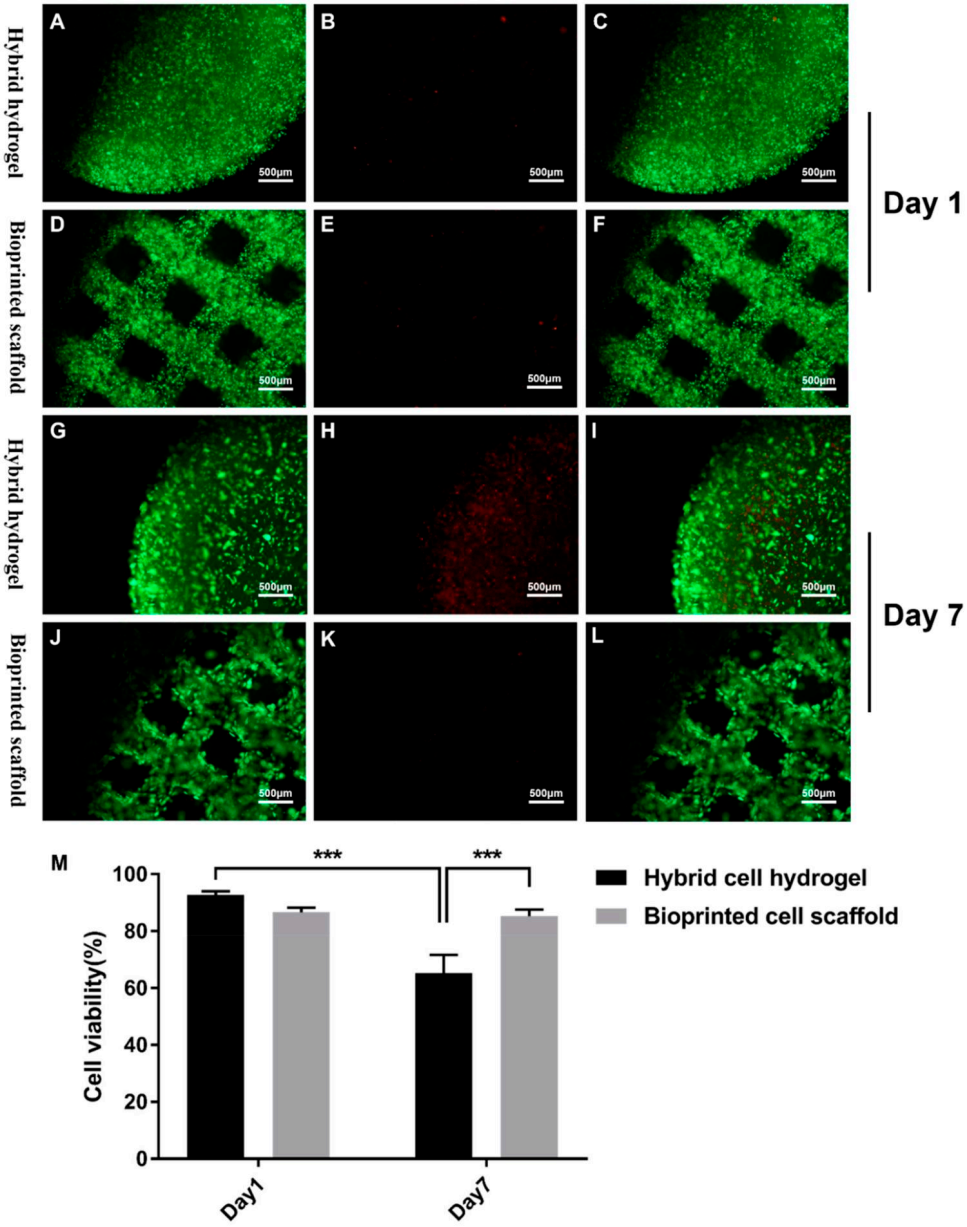
Cell viability of GSCs in 3D models. (**A**–**L**) Live/dead staining of GSCs at Day 1 and Day 7. (**M**) Cell viability of GSCs in 3D bioprinted hydrogel scaffold and hybrid hydrogel. (***p≤0.001).

**Figure 3.**
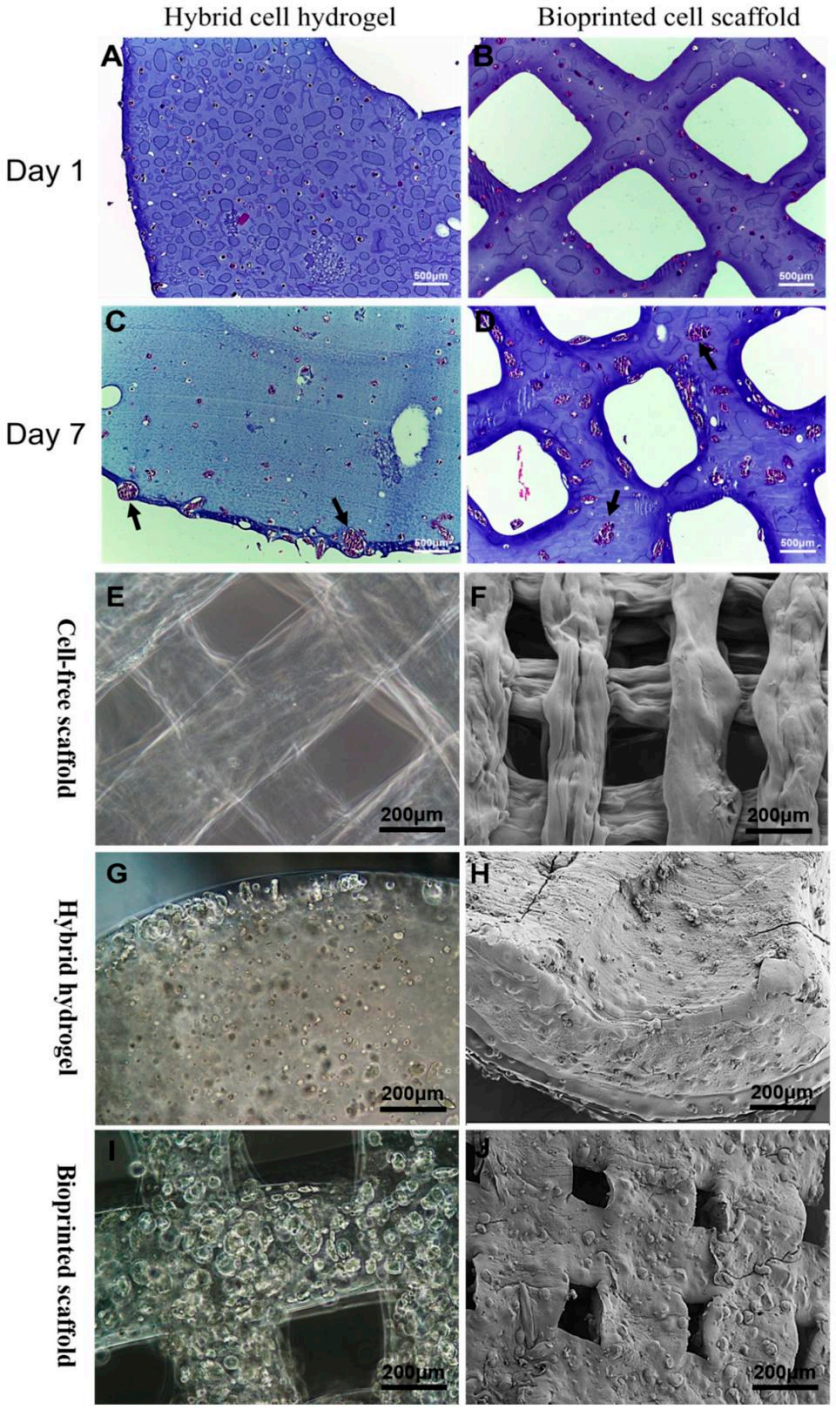
3D model under HE staining, light microscope and SEM. (**A**–**D**) HE staining of 3D bioprinted GSC-laden hydrogel scaffolds and hybrid GSC hydrogels (black arrow: GSCs cluster). (**E** and **F**) Cell-free hydrogel scaffolds at Day 7. (**G** and **H**) Hybrid GSCs hydrogels at Day 7. (**I** and **J**) 3D bioprinted GSC-laden hydrogel scaffolds at Day 7.

**Figure 4.**
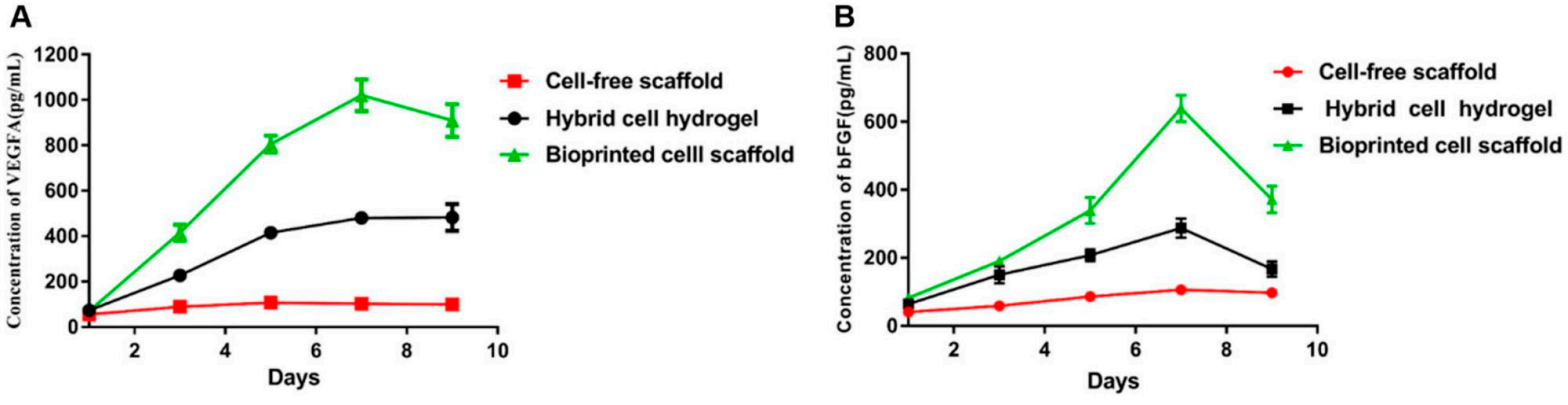
The amount of VEGFA and bFGF in co-culture dishes. (**A**) Variation of VEGFA in different 3D microenvironments. (**B**) Variation of bFGF in different 3D microenvironments.

The viability of the 3D bioprinted GSC-laden hydrogel scaffolds and hybrid GSC hydrogels was evaluated. As shown in Figure 2A–F, on the first day of culture, the cell viability of GSCs in 3D bioprinted hydrogel scaffold and hybrid hydrogel was 86.65 ± 1.64% and 92.71 ± 1.33%, respectively. Subsequently, on Day 7, the cell viability of GSCs in these two hydrogels was 85.29 ± 2.31% and 65.36 ± 6.31%, respectively (Figure 2G–L). Notably, after 7 days of culture, the viability of the GSCs in the hybrid hydrogel decreased significantly, whereas the activity of GSCs in the 3D bioprinted hydrogel scaffold remained stable and was higher than that in the hybrid hydrogel (Figure 2M). To better observe the morphology and distribution characteristics of GSCs in the 3D tumor models, HE staining and SEM were used. As shown in Figure 3A and B, the GSCs were evenly distributed in the hybrid and bioprinted hydrogels on Day 1 of the cultures. Interestingly, after seven days of culture, GSCs in both types of hydrogels formed clusters that were mainly distributed on the surface of the hybrid hydrogels, whereas GSC clusters in the bioprinted hydrogels showed a uniform distribution (Figure 3C and D). These morphological and distribution characteristics are further confirmed in Figure 3E–J. The levels of VEGFA and bFGF in the co-culture dishes were measured on Days 1, 3, 5, 7 and 9. As shown in Figure 4A and B, the quantities of VEGFA and bFGF gradually increased within 7 days, reaching a peak on the seventh day. Notably, the amounts of VEGFA and bFGF in the bioprinted group were significantly higher than those in the hybrid group.

### Bioactivity of HUVECs in co-culture system

**Figure 5.**
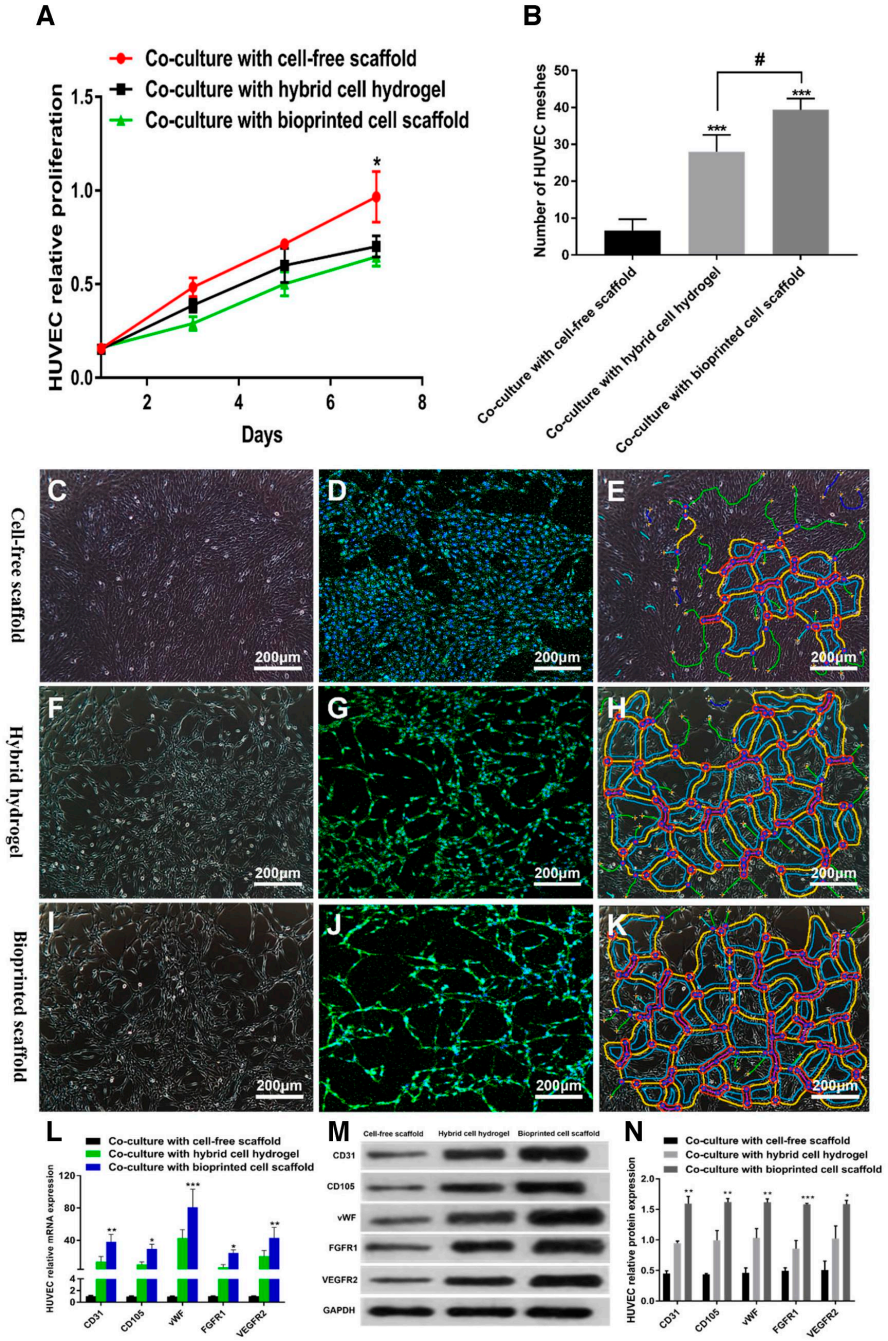
Bioactivity of HUVECs in co-culture system. (**A**) Proliferation of HUVECs co-cultured with different 3D tumor models. (**B**) Tubule-like structures were formed by HUVECs on Day 7. (**C**–**E**) HUVECs co-cultured with a cell-free scaffold on Day 7. (**F**–**H**) HUVECs co-cultured with hybrid hydrogel on Day 7. (**I**–**K**) HUVECs co-cultured with bioprinted cell scaffold on Day 7. (**L**) Relative mRNA expressions of CD31, CD105, vWF, FGFR1 and VEGFR2 in HUVECs co-cultured with bioprinted cell scaffolds were most abundant on Day 7. (**M** and **N**) The highest protein expressions of CD31, CD105, vWF, FGFR1 and VEGFR2 in HUVECs co-cultured with bioprinted cell scaffolds on Day 7. (*p≤0.05, **p≤0.01,and ***p≤0.001; #: between hybrid cell hydrogel and bioprinted cell scaffold).

Cell proliferation and morphological changes in HUVECs co-cultured with different 3D tumor models were evaluated. As shown in Figure 5A, within 7 days of culture, the HUVECs proliferated in all three microenvironments, particularly those co-cultured with cell-free scaffolds. Notably, the HUVECs formed tubule-like structures during proliferation (Figure 5C–K). Furthermore, HUVECs co-cultured with the bioprinted cell scaffolds proliferated relatively slowly but formed the largest number of tubule-like structures (Figure 5B and K). On the seventh day of co-culture, HUVECs were harvested and analyzed for the expression of genes and proteins associated with angiogenesis. As depicted in Figure 5L, the highest levels of mRNA expression for CD31, CD105, vWF, FGFR1 and VEGFR2 were observed in HUVECs that were co-cultured with bioprinted cell scaffolds. In addition, the protein expression of CD31, CD105, vWF, FGFR1 and VEGFR2 showed the same results; i.e. the protein expression in HUVECs co-cultured with bioprinted cell scaffolds was the highest (Figure 5M and N).

### Neovascularization in xenograft tumors

**Figure 6.**
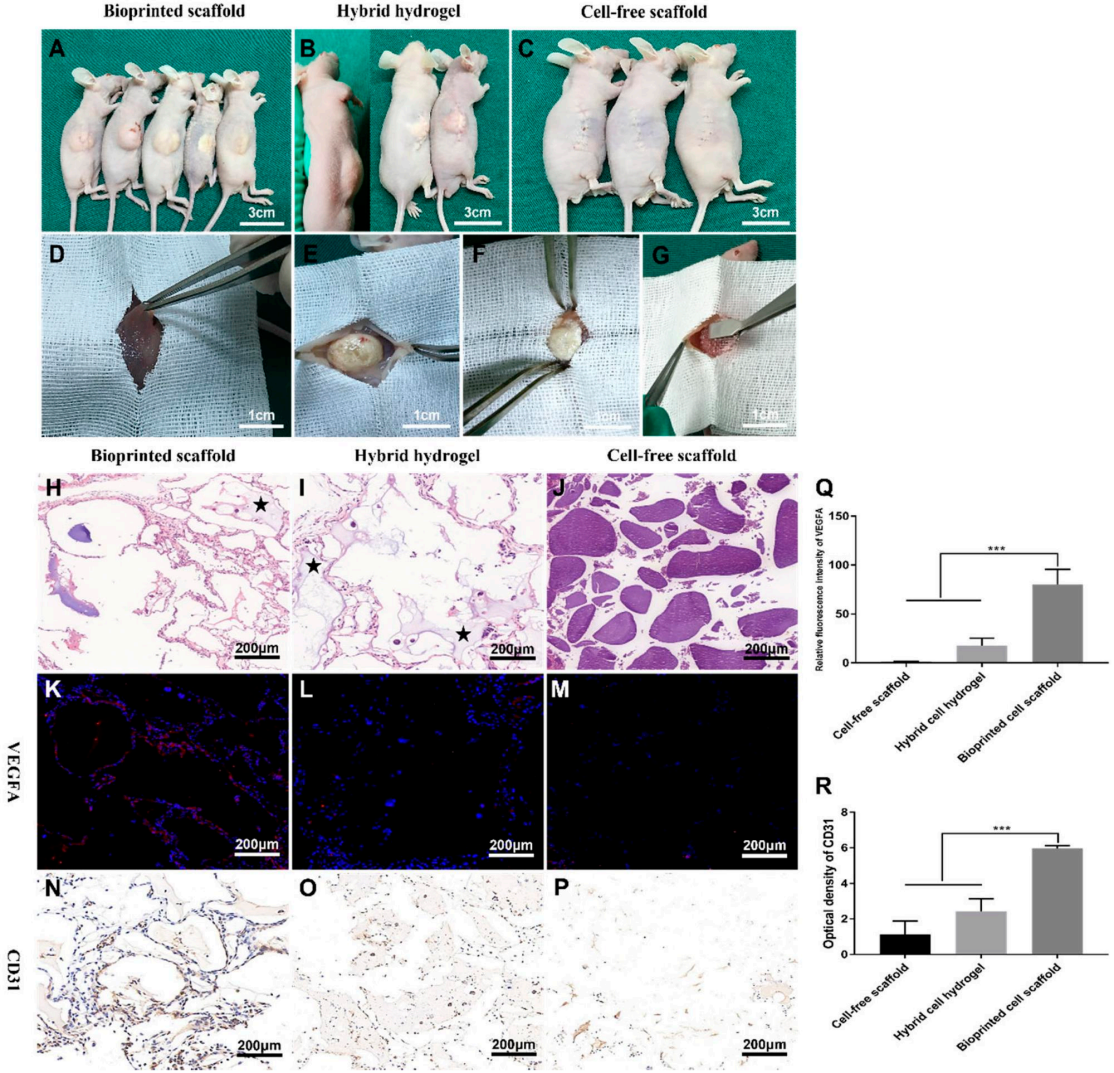
Construction and characteristics of xenograft tumors. (**A**–**C**) The xenograft tumors formed by bioprinted GSC-laden hydrogel scaffold, hybrid GSC-laden hydrogel and cell-free scaffold, respectively. (**D**) The xenograft tumor formed by bioprinted GSC-laden hydrogel scaffold was soft and had unclear boundaries with the host tissue. (**E** and **F**) The xenograft tumors formed by hybrid GSC-laden hydrogel and cell-free scaffold were tough in texture and had a clear boundary with the host tissue. (**G**) A bioprinted GSC-laden hydrogel scaffold was transplanted into the subcutaneous of nude mice. (**H** and **I**) HE staining of xenograft tumors, there was residual hydrogel inside the tumors (asterisk). (**K**–**P**) Expression of VEGFA and CD31 in xenograft tumors. (**Q** and **R**) VEGFA and CD31 were more strongly expressed in xenograft tumors formed by a bioprinted GSC-laden hydrogel scaffold. (***p≤0.001).

Xenograft tumors were constructed to analyze the vascular composition and origin of the gliomas. As shown in Figure 6G, a 3D bioprinted GSC-laden hydrogel scaffold was transplanted into the subcutaneous tissue of nude mice. The xenograft tumors were constructed after 6 weeks (Figure 6A–C). Interestingly, the xenograft tumor formed by the bioprinted GSC-laden hydrogel scaffold had a soft texture with a ‘gray-red’ color and an unclear boundary with the host tissue, similar to the growth pattern of human intracranial glioma (Figure 6D). However, the xenograft tumors formed by the hybrid GSC-laden hydrogel and cell-free hydrogel scaffold were tough, had a clear boundary with the host tissue, and appeared ‘gray-white’ (Figure 6E and F).

HE staining was used to analyze the internal morphological characteristics of the xenograft tumors. As shown in Figure 6H–I, the residual hydrogel was inside the tumors in both groups. However, the xenograft tumor formed by the hybrid cell hydrogel was more apparent. Notably, the xenograft formed by the cell-free scaffold had only a hydrogel inside and no tumor cells (Figure 6J). Significantly, both xenograft tumors expressed VEGFA (Figure 6K and L) and CD31 (Figure 6N and O). Their expression was significantly enhanced in tumors formed by the bioprinted GSC-laden hydrogel scaffold (Figure 6Q and R).

**Figure 7.**
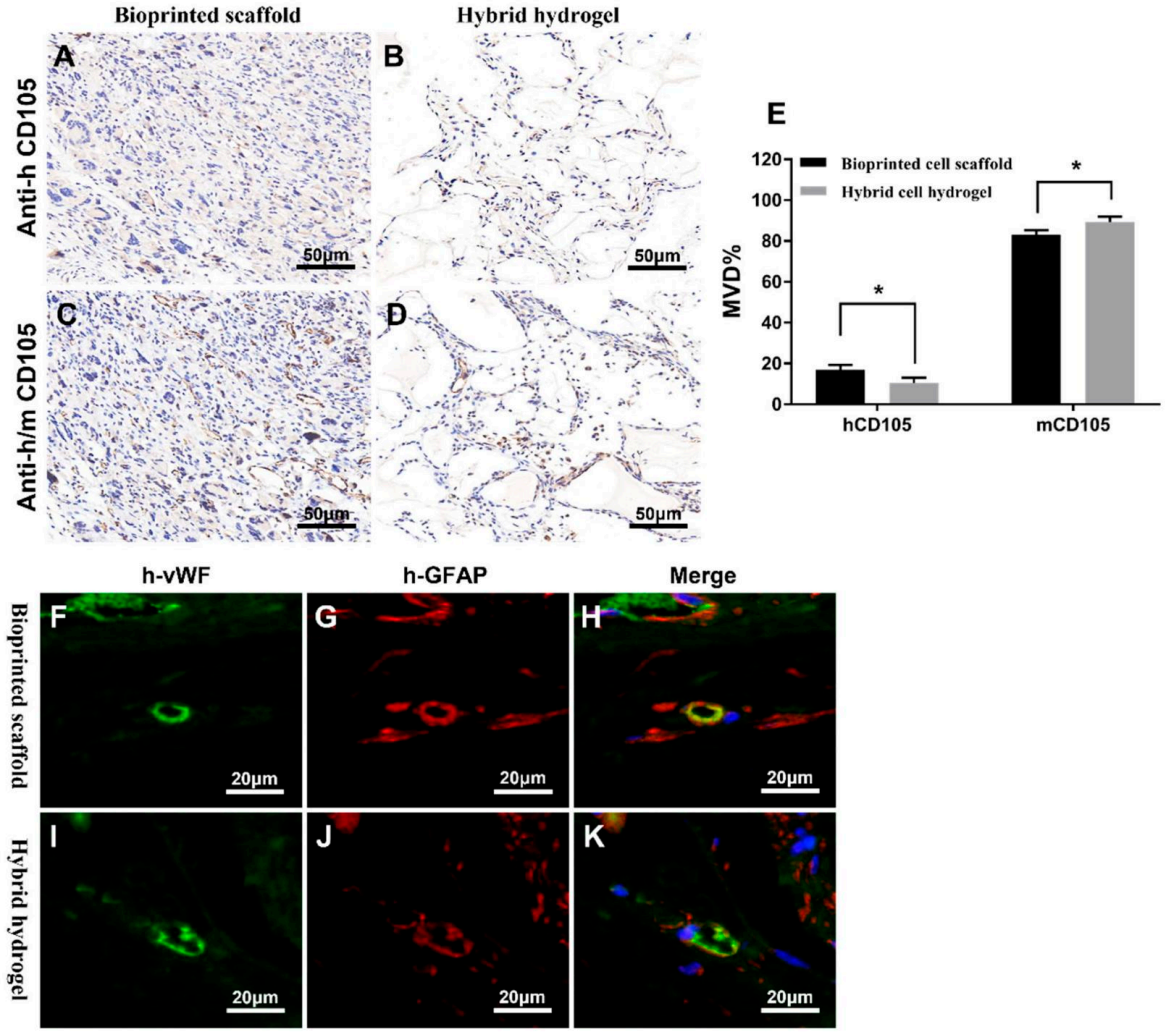
Expression of CD105^+^ cells and co-expression of h-vWF and h-GFAP in xenograft tumors derived from bioprinted GSC-laden hydrogel scaffold and hybrid GSC hydrogel. (**A** and **B**) Anti-human CD105 was used to evaluate xenograft tumors. (**C** and **D**) Anti-human/mouse CD105 was used to evaluate xenograft tumors. (**E**) MVD% of human (h) and mouse (m) CD105^+^cells in xenograft tumors. (**F**–**K**) Double immunofluorescence staining was used to label anti-human vWF (green) and anti-human GFAP (red). Nuclei are counterstained blue. (*p≤0.05).

As shown in Figure 7A–D, human endothelial-like cells labeled with human-specific anti-CD105 and murine endothelial-like cells labeled with anti-human/mouse CD105 were expressed in the xenograft tumors formed by the bioprinted GSC-laden hydrogel scaffold and hybrid GSC hydrogel, respectively. However, xenograft tumors formed by bioprinted GSC-laden hydrogel scaffolds contained more human endothelial cell-like cells than the hybrid GSC hydrogel-derived xenograft tumors. Furthermore, 16.98 ± 2.34% of CD105^+^ cells in xenograft tumors formed by bioprinted GSC-laden hydrogel scaffold were from a human being, whereas only 10.50 ± 2.51% of CD105^+^ cells in xenograft tumors formed by hybrid GSC hydrogel were from a human being (Figure 7E).

As shown in Figure 7F–K, the presence of human endothelium-vascular lumen structures in GSC-derived xenograft tumors was demonstrated by the co-expression of anti-human vWF and GFAP.

## Discussion

This study constructed a bioprinted GSC-laden hydrogel scaffold, hybrid GSC hydrogel and cell-free hydrogel scaffold. A co-culture dish was designed and manufactured to better observe the effects of GSCs in the 3D model on the surrounding endothelial cells. This dish consisted of two concentric circles: an outer and an inner dish. The inner dish communicates with the outer dish through the side holes. Herein, the cell viability of GSCs in 3D bioprinted hydrogel scaffold (86.65 ± 1.64%) and hybrid hydrogel (92.71 ± 1.33%) was higher on Day 1. However, with an increase in the incubation time, the viability of the GSCs in the hybrid hydrogel decreased significantly, and the activity of the GSCs in the bioprinted hydrogel scaffold remained stable. We hypothesize that this is owing to the gridded shape of the bioprinted cell-laden hydrogel scaffolds, which facilitates the availability of nutrients to the cells in different areas. However, the hybrid cell hydrogel model does not have a mesh; therefore, the closer the hydrogel center, the fewer nutrients the cell receives. Furthermore, cluster-like GSCs were found in both 3D hydrogel models, but the difference was that cluster-like GSCs were uniformly distributed in the bioprinted hydrogel scaffold and only distributed on the surface of the hybrid hydrogel model. These results indicated that bioprinted hydrogel scaffolds are beneficial for the survival and maintenance of GSC biological characteristics [16]. Previous studies found that glioma cells secreted VEGFA in a 3D bioprinted hydrogel model [4]. In this study, HUVECs were co-cultured with GSCs in different 3D microenvironments, and we found that VEGFA and bFGF levels in bioprinted hydrogel scaffolds were significantly higher than those in the hybrid hydrogel model on the seventh day. We believe that GSCs in bioprinted hydrogel scaffolds have better biological activity and secrete more vascular growth factors when co-cultured with HUVECs.

Studies have shown that VEGFA and bFGF increase vascular permeability and are involved in physiological and tumor-induced vascularization [17–19]. This study evaluated cell proliferation and morphological changes in HUVECs co-cultured with different 3D tumor models. Notably, within 7 days of co-culture, HUVECs proliferated in all three microenvironments, particularly those co-cultured with cell-free scaffolds. Interestingly, we found that the HUVECs formed tubule-like structures during proliferation and that the lower the proliferation rate, the more tubule-like structures were formed. We hypothesized that this was because the 3D bioprinted GSC-laden hydrogel scaffold secreted more vascular growth factors to facilitate the formation of tubule-like structures by HUVECs. Furthermore, CD31, CD105, vWF, FGFR1 and VEGFR2 expression levels, which are associated with angiogenesis in HUVECs, were evaluated. Our results demonstrated that the gene and protein expression of these markers in HUVECs were most abundant when co-cultured with 3D bioprinted cell scaffolds. We hypothesized that VEGFA and bFGF secreted by 3D bioprinted GSC-laden scaffold promote the expression of vascularization-related markers in HUVECs. Autocrine and paracrine interactions between GSCs and HUVECs may enhance the expression of these markers [20].

To further investigate the involvement of 3D-GSCs in tumor angiogenesis *in vivo*, bioprinted GSC-laden hydrogel scaffolds, hybrid GSC hydrogels and cell-free hydrogel scaffolds were transplanted into the subcutaneous tissues of nude mice. Remarkably, the characteristics of the xenograft tumor formed by bioprinted GSC-laden hydrogel scaffolds were similar to those of human intracranial glioma, showing a soft texture, grayish-yellow color and unclear boundaries with the surrounding tissue [21]. However, xenografts derived from the hybrid GSC-laden hydrogel and cell-free hydrogel scaffold were tough, grayish-white, and had clear boundaries with the host tissue. Furthermore, the hydrogels remained in both xenograft tumors, particularly those formed by cell-free hydrogel scaffolds. These results indicated that the xenograft tumor formed by the bioprinted GSC-laden hydrogel scaffold was more consistent with the morphology of glioma *in vivo*, which was helpful for the study of tumor angiogenesis mechanisms. CD31 and VEGFA are markers of angiogenesis that reflect the degree of neovascularization [22, 23]. In this study, among the three types of xenografts, the expressions of CD31 and VEGFA derived from the bioprinted GSC-laden hydrogel scaffold were the most significant, indicating abundant neovascularization. Notably, murine and human endothelial-like cells were found in the xenograft tumors formed by bioprinted GSC-laden hydrogel scaffolds and hybrid GSC-laden hydrogels. More specifically, 16.98 ± 2.34% of CD105^+^ cells in xenograft tumors formed by bioprinted GSC-laden hydrogel scaffold were of human origin, which was significantly higher than that of xenograft tumors formed by hybrid GSC hydrogel, where only 10.50 ± 2.51% of CD105^+^ cells were derived from humans. This not only indicated that glioma cells could directly participate in tumor angiogenesis but also that in xenograft tumors, the participation rate of bioprinted 3D glioma cells was higher. More importantly, we found a portion of the vascular lumen-like structure composed of endothelial/glial phenotypic (human-vWF^+^/human-GFAP^+^) cells. This further suggests that glioma cells were involved in glioma neovascularization via direct transdifferentiation and/or fusion with endothelial cells.

## Conclusion

In this study, 3D bioprinted GSC-laden hydrogel scaffolds, hybrid GSC hydrogels and cell-free hydrogel scaffolds were developed to investigate the mechanism of glioma angiogenesis. We discovered that the bioactivity of GSCs in a 3D bioprinted GSC-laden hydrogel scaffold was the most effective and stable, with the highest levels of VEGFA and bFGF in the microenvironment. When a 3D bioprinted GSC-laden hydrogel scaffold, hybrid GSC hydrogel and cell-free hydrogel scaffold were co-cultured with HUVEC, the expression of markers associated with angiogenesis was the most abundant when co-cultured with 3D bioprinted GSC-laden hydrogel scaffold. Furthermore, the xenograft tumor formed by the bioprinted GSC-laden hydrogel scaffold was similar to human glioma in color, texture and vascularization. Notably, the number of human CD105^+^ cells was significantly higher in xenograft tumors formed using bioprinted GSC-laden hydrogel scaffolds, and human endothelium-vascular lumen structures were observed in both types of 3D-GSC-derived tumors.

In summary, these findings demonstrate that GSCs have better biological activity in 3D bioprinted models than hybrid GSCs, which have excellent angiogenesis effects *in vitro* and participate in tumor angiogenesis *in vivo* through direct transdifferentiation and/or fusion of endothelial cells. We believe that the 3D bioprinted GSC-laden hydrogel scaffold is a suitable model to mimic the glioma microenvironment and can be a candidate for studying tumor angiogenesis and developing targeted drugs in future research programs.
